# Impact of village health volunteer support on postnatal depressive symptoms in the remote rural areas of Lao People’s Democratic Republic: a cross-sectional study

**DOI:** 10.1186/s41182-021-00316-0

**Published:** 2021-03-30

**Authors:** Noriko Toyama, Inthanomchanh Vongphoumy, Manami Uehara, Chika Sato, Futoshi Nishimoto, Kazuhiko Moji, Tiengkham Pongvongsa, Kokoro Shirai, Tomomi Takayama, Misuzu Takahara, Yoko Tamashiro, Yumiko Endo, Sengchanh Kounnavong, Jun Kobayashi

**Affiliations:** 1 School of Health Sciences, Faculty of Medicine, University of the Ryukyus, Okinawa, Japan; 2 Savannakhet Provincial Health Department, Savannakhet, Lao People’s Democratic Republic; 3 Department of Global Health, Graduate School of Health Sciences, University of the Ryukyus, Okinawa, Japan; 4 Asia Health and Education Fund, Tokyo, Japan; 5 Nagasaki University School of Tropical Medicine and Global Health, Nagasaki, Japan; 6 Department of Public Health, Graduate School of Medicine, Osaka University, Osaka, Japan; 7 Lao Tropical and Public Health Institute, Ministry of Health, Vientiane, Lao People’s Democratic Republic

**Keywords:** Postnatal depressive symptoms, Village health volunteer, Social support, Cross-sectional study

## Abstract

**Background:**

Village health volunteers (VHVs) are responsible for providing primary care in the communities of Laos. Unlike other districts, in Xepon more than 90% of VHVs are male and therefore experience difficulties interacting with pregnant women. To improve outreach to pregnant women, especially among ethnic minorities, a new project was implemented by local municipalities in 2017: newly selected female VHVs were paired to work with existing male VHVs. The objective of this study was to compare the postnatal depressive symptoms of ethnic minority mothers supported by pair-VHVs and single-VHVs in remote rural areas of Lao People’s Democratic Republic (PDR).

**Methods:**

A cross-sectional study was conducted in March 2019. Mothers who had delivered a baby within 1 year preceding the study were recruited from 36 villages. Of the 305 mothers, 227 responded. The questionnaires included (1) demographic characteristics such as age, economic status, and birth experience; (2) self-decision to go to a health center/hospital to receive antenatal care; (3) type of VHVs (pair or single), support, and information from VHVs during pregnancy, support from husband and relationship with husband; (4) the Edinburgh Postnatal Depression Scale (EPDS). A Mann-Whitney *U* test, chi-square test, and multiple linear regression analysis were performed. Ethical approval was obtained from the University of the Ryukyus and National Ethics Committee for Health Research of Lao PDR.

**Results:**

The average total EPDS score was 5.5 among mothers supported by pair-VHV and 7.0 among mothers supported by single-VHV. Results of the multiple linear regression analysis showed that the EPDS score was significantly lower among mothers supported by pair-VHV (*β*=−1.18, *p* <0.05) even after adjusting for economic and biological factors.

**Conclusions:**

Mothers supported by pair-VHV had a significantly lower EPDS score than those supported by single-VHVs in this study area, suggesting that the support of male and female VHV pairs contributed to improving mental health status among ethnic minority mothers in remote rural areas of Lao PDR. Expanding the program to train female VHVs working with male VHVs is necessary for improving maternal and child health in a rural district of Lao PDR.

## Background

The impact of maternal mental health problems on infants and children has been identified mostly in terms of psychosocial and emotional development [1–3]. The effects of maternal mental health on child abuse and neglect have also been reported [4, 5]. Previous studies show that the prevalence of maternal mental disorders in low- and middle-income countries (LMICs) is higher than that in high-income countries (HICs) [6]. The World Health Organization (WHO) attributes this high prevalence of maternal mental disorders in LMICs to adolescent pregnancy, unsupportive marital relationships, nulliparity, poverty and lack of financial resources, lack of practical support, illiteracy, minimal assistance, poverty, problematic relationships with in-law families, and so on [6].

Previous studies reported that social support has a positive effect on maternal mental health status in both HICs and LMICs [7–9]. According to the WHO, community health workers (CHWs) are people engaged in primary health care in the community on behalf of medical personnel [10]. CHWs are effective in delivering a variety of services and interventions including providing basic health care, health education, and promoting the uptake of health services in rural- and low-income settings. Therefore, CHWs may be one of the resources needed to improve maternal mental health status in LMICs.

Lao PDR is a LMIC in Asia, and still faces high maternal and infant mortality, even though maternal mortality has successfully declined from 1660/100,000 live births in 1990 to 197/1,000,000 in 2015 [11, 12]. Despite the considerable recent progress in maternal and child health, health disparities such as those between urban and rural areas, the rich and poor, and major and minor ethnic groups have increased. Therefore, it is necessary to pay attention to pregnant women in rural and ethnic minorities.

In Lao PDR, village health volunteers (VHVs) work as CHWs and play an important role in the community. The main activities of VHVs include helping health workers with outreach activities, providing health education to communities, providing basic primary care in remote areas, referring patients to health care facilities, facilitating prenatal care clinics at health care facilities, and conducting community-based surveillance for vital events and malaria in villages with endemic malaria [13]. VHVs in each village were selected according to criteria such as possessing good health, completion of at least a primary level of education, and willingness to work on a voluntary basis. In Xepon district, VHVs play an important role in providing primary health care; however, they are mostly male. Many women are not qualified to become VHVs because of the high dropout rate in early primary school and illiteracy. Therefore, male VHVs sometimes face challenges in identifying newly pregnant women and communicating with them.

To promote maternal and child health, a community-based approach was launched in September 2017 in Xepon district. New female VHVs from each village were selected in 19 villages and received training related to recognizing the signs of danger during pregnancy, importance of health checkups for pregnant women, and collaboration with health centers/hospital staff. Thereafter, female VHVs partnered with male VHVs in the same villages and began joint activities in the community. It was hoped that working in male-female pairs would improve outreach to women who are ethnic minorities and live in poor rural areas, and would have a positive impact on postnatal depressive symptoms. However, no study to date has examined the association between VHVs working as pairs and postnatal depressive symptoms.

## Methods

### Study aim, design, and setting

This study aimed to compare the postnatal depressive symptoms of mothers supported by pair-VHVs and single-VHVs in remote rural areas in Lao PDR. Therefore, a cross-sectional study was conducted in Xepon district, which is located in the western area of Savannakhet province. Xepon district is a rural and mountainous area on the Vietnamese border, about 600 km from the capital Vientiane. There are 88 villages in the district, which houses a total population of about 56,000 people [14]. Residents use their own distinctive languages and have limited formal education, especially girls. In Savannahket province, the overall net enrollment rate in primary school is 90.3% (88.7% for females). However, because of the economic and geographic situation, in 2012, the enrollment rate was only 64.2% for females in Xepon district [15]. Dropout from primary schools among girls is high. Only 50% of female students are promoted to grade 5 [15]. Some women hesitate to use antenatal care (ANC) services because they do not understand Lao and thus experience difficulties in communicating with health staff [16].

We targeted all 19 villages where VHVs work as pairs and selected another 19 villages where VHVs work alone in Xepon district. The selection criteria were a similar population size and distance to a health facility. Ultimately, of the 19 selected villages, 17 were targeted because we were unable to contact the village leader or VHV of two villages.

### Study participants

**Fig. 1:**
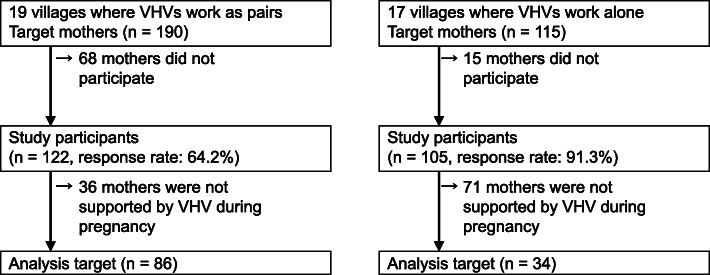
Flow chart of the study participants

In each village, we recruited mothers who had delivered a baby 1 year preceding data collection, had lived in the same village for more than 1 year, and were aged 15 years or more. As Fig. 1 shows, 122 mothers of 190, from 19 villages where VHVs work as pairs and 105 mothers out of 115 from 17 villages where VHVs work alone, participated in the study. We excluded 36 and 71 mothers, respectively, since they were not supported by VHVs during pregnancy. As a result, 86 and 34 mothers, respectively, were included in the data analysis. All participants provided informed consent verbally.

### Data collection

Prior to the research, we trained research assistants on the interview method and ethical issues. Data were collected in March 2019 via face-to-face interviews with structured questionnaires. A Lao language questionnaire was used in this study, and VHVs in each village supported interpretation from the Lao language to the local language when mothers had difficulty in understanding the questionnaire.

### Variables

#### Outcome variable

The status of postnatal depressive symptoms was measured using the Edinburgh Postnatal Depression Scale (EPDS). The EPDS consists of 10 items and is widely used to screen postpartum depression [17]. The EPDS was developed from a Western paradigm; however, its reliability and validity have been tested, and the EPDS has been used widely in Asian countries such as Japan and China [18, 19]. The scoring system is 0 to 3 points for one question, and the total score is 0 to 30 points for one participant. The cut-off point of the EPDS was different in each country, and there has not been research providing a cut-off point in Lao PDR. Therefore, we used the EPDS score as a continuous variable in our analysis. The Lao language version of the EPDS was obtained from The Health Translations Directory, an initiative of the Victorian Government of the Australian state of Victoria (https://www.healthtranslations.vic.gov.au). This version was pretested involving six mothers with 1-year old children living in the study area. Following the pretest, the wording was modified in a few instances by health staff working at Xepon district.

#### Predictor variables

The predictor variables were selected based on the following risk factors of mental health problems in the WHO report: (1) biological characteristics such as age and parity, (2) economic status such as possession of a motorbike, and (3) social factors such as the self-decision to go to a health center/hospital to receive ANC, and type of VHV support (pair or single).

#### Other variables

To show differences between the activities of pair-VHVs and single-VHVs, four types of questions were collected. (1) Support from VHVs during pregnancy consisted of seven items using a 5-point Likert scale. (2) Information given by VHVs during pregnancy consisted of nine items using a 4-point Likert scale. These two types of questions were developed by the authors and local health staff considering the local situation. (3) To assess husbands’ support during pregnancy, three questions applied in previous research were used for analysis. Two of these questions were related to a reduction in workload during pregnancy and one question has been commonly used to assess male partner involvement [20, 21]. Each of these three questions used a 5-point Likert scale. (4) The participants’ relationships with their husbands consisted of four items requiring yes or no responses. Provision and reception of emotional support and instrumental support between respondents and husbands were used for analysis. These four items were selected and applied from components of health-related community social capital in consideration of the purpose of this study [22].

### Data analysis

Frequency distributions were generated for the predictor variables. A Mann-Whitney *U* test was performed for the bivariate association between the EPDS score, VHV support, information from VHV during pregnancy, husband’s support during pregnancy, and type of VHV. A *t* test was used to determine the bivariate association between the total EPDS score and type of VHV. A chi-square test was performed for the bivariate association between relationship with husband and type of VHV. Furthermore, a multiple linear regression analysis was conducted for the multivariate association of postnatal depressive symptoms and pair-VHV support. Model 1 included social factors as the independent variable and EPDS as the dependent variable. Economic factors were added as independent variables in model 2 and biological factors in model 3. SPSS version 26 was used for this analysis. Statistical significance was set as *p*<0.05.

### Ethics

The study adhered to the rules and regulations of the Ethics Review Board/Institutional Review Board, in accordance with the World Medical Association Declaration of Helsinki. Ethical approval was obtained from the National Ethics Committee for Health Research, Ministry of Health, Lao PDR, and the University of the Ryukyus.

## Results

### Socio-demographic and economic characteristics of study participants

Approximately three-quarters (70.9% of mothers supported by pair-VHV, 82.4% of mothers supported by single-VHV) were aged between 20 and 34 years. The average age of mothers supported by pair-VHV was 24.2 years, and 25.5 years for mothers supported by single-VHV (Table 1). The majority (69.5%) belonged to the Makong/Tree ethnic group. More than 60% of the participants (62.4% and 70.6%, respectively) had not received formal education. Approximately 60% of the participants (66.3% and 58.8%, respectively) had motorbikes. More than 70% of the participants (72.1% and 79.4%, respectively) were multiparas. Less than half the participants (30.2% and 44.1%, respectively) made the self-decision to go to a health center/hospital to receive ANC.

**Table 1:** Socio-demographic and economic characteristics of study participants (*n* = 120)

	Pair-VHV *n*=86		Single-VHV *n*=34	
	Number	(%)	Number	(%)
Age				
< 20 years	18	(20.9)	4	(11.8)
20–34 years	61	(70.9)	28	(82.4)
34 years <	7	(8.1)	2	(5.9)
Mean (SD) min-max	24.2 (±6.1)	16-43	25.5 (±6.1)	15-45
Ethnicity				
Makong/Tree	54	(62.8)	21	(61.8)
Phouthai	20	(23.3)	4	(11.8)
Others	12	(14.0)	9	(26.4)
Education				
No formal education	53	(62.4)	24	(70.6)
Primary (1–5 years)	24	(28.2)	9	(26.5)
Secondary or above (< 6 years)	8	(9.4)	1	(2.9)
Economic status				
Possession of a motorbike	57	(66.3)	20	(58.8)
Parity				
Primiparas	24	(27.9)	7	(20.6)
Multiparas	62	(72.1)	27	(79.4)
Self-decision to go to a health center/hospital to receive ANC				
Yes	26	(30.2)	15	(44.1)
No	60	(69.8)	19	(55.9)
EPDS				
7<	28	(32.6)	18	(52.9)
8<	21	(24.4)	13	(38.2)
9<	13	(15.1)	8	(23.5)
10<	11	(12.8)	8	(23.5)
11<	11	(12.8)	6	(17.6)
12<	6	(7.0)	5	(14.7)

*SD* standard deviation

### Prevalence of postpartum depressive symptoms

Cronbach’s coefficient alpha of EPDS was 0.66. In total, 28 (32.6%) and 18 (52.9%) mothers supported by pair-VHVs and by single-VHVs, respectively, had a total score of more than 7 on the EPDS. Furthermore, 21 (24.4%, pair-VHV) and 13 (38.2%, single-VHV) mothers had a total score of more than 8 on the EPDS, 13 (15.1%) and 8 (23.5%) scored more than 9, 11 (12.8%) and 8 (23.5%) scored more than 10, and 11 (12.8%) and 6 (17.6%) scored more than 11.

### Bivariate association between type of VHV and EPDS score

Regarding the EPDS score, the average of the total score was 5.5 and 7.0, respectively (Table 2). The highest score among mothers supported by single-VHVs (1.1) was for the EPDS item: “I have been so unhappy that I have had difficulty sleeping.” The highest score among mothers supported by pair-VHVs (1.1) was for the item: “I have looked forward with enjoyment to things.” Scores for the two items “I have blamed myself unnecessarily when things went wrong” and “I have been so unhappy that I have had difficulty sleeping” were significantly lower among mothers supported by pair-VHVs.

**Table 2:** Bivariate association between type of VHV and EPDS score (*n* = 120)

	Pair-VHV *n*=86		Single-VHV *n*=34		*p*
	M	(SD)	M	(SD)	
1. I have been able to laugh and see the funny side of things ^a^	0.6	(0.9)	0.9	(1.0)	0.15
2. I have looked forward with enjoyment to things ^a^	1.1	(1.2)	0.8	(1.1)	0.17
3. I have blamed myself unnecessarily when things went wrong ^a^	0.9	(1.0)	1.0	(1.0)	<0.01
4. I have been anxious or worried for no good reason ^a^	0.5	(0.8)	1.0	(1.0)	0.46
5. I have felt scared or panicky for no good reason ^a^	0.6	(0.9)	0.8	(0.9)	0.23
6. Things have been getting on top of me ^a^	0.5	(0.7)	0.5	(0.7)	0.77
7. I have been so unhappy that I have had difficulty sleeping ^a^	0.6	(0.9)	1.1	(1.0)	0.01
8. I have felt sad or miserable ^a^	0.3	(0.5)	0.4	(0.6)	0.29
9. I have been so unhappy that I have been crying ^a^	0.4	(0.6)	0.5	(0.6)	0.20
10. The thought of harming myself has occurred to me ^a^	0.0	(0.3)	0.0	(0.2)	0.86
Total^b^	5.5	(3.9)	7.0	(4.5)	0.08

^a^Mann-Whitney *U* test

^b^*T* test

### Multivariate association of postnatal depressive symptoms and pair-VHV support

The results of the multiple linear regression analysis showed that mothers supported by pair-VHVs had significantly lower scores on the EPDS than mothers supported by single-VHVs (*β* = −0.19, *p <0.05*) in model 1 (Table 3). In model 2, the association between postnatal depressive symptoms and pair-VHVs support remained statistically significant (*β* = −0.18, *p <0.05*). In model 3, the association between postnatal depressive symptoms and pair-VHV support remained statistically significant (*β* = −0.19, *p <0.05*). Apart from the association with type of VHV support, the self-decision to go to a health center/hospital to receive ANC (*β* = −0.26, *p <0.01*), and possession of a motorbike (*β* = −0.25, *p <0.01*) were also significantly associated with postnatal depressive symptoms in model 3.

**Table 3:** Multivariate association of postnatal depressive symptoms with type of VHV support (*n* = 120)^a^

	Model 1			Model 2			Model 3		
	***B***	***β***		***B***	***β***		***B***	***β***	
Social factors									
Type of VHV support^b^	−1.77	−0.19	*	−1.63	−0.18	*	−1.76	−0.19	*
Self-decision^c^	−2.31	−0.27	**	−2.24	−0.26	**	−2.24	−0.26	**
Economic factor									
Possession of a motorbike^d^				−1.86	−0.22	*	−2.10	−0.25	**
Biological factors									
Age							−0.03	−0.05	
Parity^e^							−1.52	−0.16	
R2		0.05			0.08			0.10	

^a^According to multiple linear regression analysis

^b^Type of VHV support: pair-VHV support = 1, single-VHV support = 0

^c^Self-decision: yes=1, no=0

^d^Possession of a motorbike: yes=1, no=0

^e^Parity: multiparas=1, primiparas=0

*p<0.05

**p<0.01

### Bivariate association between participants’ type of VHV and VHV support during pregnancy

The results of the Mann-Whitney *U* test showed that compared to participants supported by single-VHVs (*p* < 0.01), those supported by pair-VHVs had a significantly higher level of VHV support during pregnancy, as reflected by the following items: “VHV checked danger signs during pregnancy,” “VHV measured my blood pressure,” and “VHV came with me to the ANC visit” (Table 4).

**Table 4:** Bivariate association between type of VHV and VHV support during pregnancy (*n*=120)^a^

	Pair-VHV		Single-VHV		*p*
	*n*=86		*n*=34		
	M	(SD)	M	(SD)	
1. VHV checked danger signs during pregnancy	4.3	(0.8)	2.8	(1.4)	<0.01
2. VHV measured my blood pressure	4.4	(0.9)	2.4	(1.5)	<0.01
3. VHV encouraged me to go to ANC	4.4	(0.8)	4.1	(1.0)	0.12
4. VHV came with me to ANC visit	3.6	(1.4)	2.4	(1.4)	<0.01
5. VHV stayed and helped me while health center staff provided ANC in my community	4.3	(0.7)	3.9	(1.3)	0.25
6. VHV talked to my husband/family about important things to know during pregnancy	4.2	(1.0)	3.9	(1.1)	0.28
7. VHV offered me transportation to a health center when necessary	3.2	(1.6)	2.9	(1.4)	0.15

*M* mean, *SD* standard deviation

^a^According to Mann-Whitney *U* test

### Bivariate association between type of VHV and information from VHV during pregnancy

The results of the Mann-Whitney *U* test showed that compared to participants supported by single-VHVs, those supported by pair-VHVs received significantly more information from a VHV during pregnancy on the “necessity of receiving ANC,” “importance of facility-based delivery,” and “danger signs during pregnancy” (*p* < 0.05) (Table 5).

**Table 5:** Bivariate association between type of VHV and information given by VHV during pregnancy (*n*=120)^a^

	Pair-VHV		Single-VHV		*p*
	*n*=86		*n*=34		
	M	(SD)	M	(SD)	
1. Necessity of receiving ANC	3.5	(0.6)	3.0	(1.0)	0.03
2. Importance of facility-based delivery	3.5	(0.5)	3.1	(0.9)	0.02
3. Danger signs during pregnancy	3.4	(0.6)	2.8	(1.1)	<0.01
4. The context of ANC services	3.5	(0.6)	3.2	(0.9)	0.14
5. Importance of support from husband	3.4	(0.7)	3.1	(0.9)	0.09
6. Advice on preparing for birth	3.4	(0.6)	3.1	(1.0)	0.19
7. Advice on delivering the baby	3.3	(0.6)	3.0	(1.0)	0.15
8. Advice on breastfeeding	3.3	(0.7)	3.1	(0.8)	0.40
9. Advice on raising children	3.3	(0.6)	3.1	(0.9)	0.26

*M* mean, *SD* standard deviation

^a^According to Mann-Whitney *U* test

### Bivariate association between type of VHV and husband’s support and relationships

The results of the Mann-Whitney *U* test showed that compared to participants supported by single-VHVs, those supported by pair-VHVs had a significantly higher level of their husband’s support, as reflected in the following items: “He wanted me to stay away from farming and hard labor” (*p* < 0.05) (Table 6).

**Table 6:** Bivariate association between type of VHV and husband’s support and relationship (*n* = 120)

	Pair-VHV		Single-VHV		*P*
	*n*=86		*n*=34		
Husband’s support during pregnancy	M	(SD)	M	(SD)	
He wanted me to stay away from farming and hard labor ^a^	4.1	(1.0)	3.6	(1.1)	0.02
He took care of the older children more often ^a^	4.1	(1.2)	3.8	(1.1)	0.08
He took me to a health facility to receive ANC^a^	4.1	(1.2)	4.2	(1.1)	0.97
Relationship with husband	n	(%)	N	(%)	
My husband listens to my concerns and complaints ^b^	62	(72.1)	19	(55.9)	0.09
I listen to my husband’s concerns and complaints ^b^	56	(65.1)	16	(47.1)	0.07
My husband looks after me when I am sick and confined to a bed for a few days ^b^	80	(93.0)	28	(82.4)	0.08
I look after my husband when he is sick and confined to a bed for a few days ^b^	73	(84.9)	26	(76.5)	0.27

*M* mean, *SD* standard deviation

^a^Mann-Whitney *U* test

^b^Chi-square test

The results of the chi-square test did not demonstrate statistical significance; however, compared to participants supported by single-VHVs, those supported by pair-VHVs reported having a better relationship with their husband, as reflected in the following items: “My husband listens to my concerns and complaints,” “I listen to my husband’s concerns and complaints,” “My husband looks after me when I am sick and confined to a bed for a few days,” and “I look after my husband when he is sick and confined to a bed for a few days” (Table 6).

## Discussion

This study showed that mothers supported by pair-VHVs had a better mental health status than mothers supported by single-VHVs in a rural district of Lao PDR even after adjusting for potential confounding factors. This result supports the results of previous studies. For example, a study conducted in Japan reported that those who received continued support from nurses and midwives showed better mental health in the postpartum period [7]. Another study conducted in the USA demonstrated that social support such as that perceived from a partner, and family/friends was important to the physical and mental health of new mothers [8]. An Ethiopian study further showed that low social support including family support, support from a friendship network, and help from a spouse were independent predictors of postpartum depressive symptoms [9].

According to the results of this study, several types of support and information were significantly higher among mothers supported by pair-VHVs. In particular, it was very helpful for pregnant women when a VHV accompanied them to ANC visits in the area. Sato reported that one barrier for pregnant women to access health care facilities was “language difficulty,” since their ethnicity differed from that of the health workers in the area [16]. Most of the women in our study belonged to the Makong or Tree tribe and had not received formal education; therefore, conversation in Lao was difficult for them. The training of pair-VHVs to emphasize the importance of ANC and collaboration with health centers/hospital staff was a success. Especially in this study area, women in their mid-teens tend to marry men who live in villages far from their hometown, which means that they cannot easily ask for help from their own mother/family and often do not have friends in the neighborhood. They also tend not to have life and social skills because of their low level of education and young age. In addition, women find it difficult to ask for support from single-VHVs because most are male and engaged in various types of work in the area.

Furthermore, the results of this study showed that participants supported by pair-VHVs received a significantly higher level of support from their husbands and had a better relationship with their husbands than those supported by single-VHVs. A previous study showed that husband’s support was an important factor in improving the mental health status of mothers [8, 9]. As such, support from pair-VHVs influences not only the mental health status of mothers, but also improves the support received from their husbands and enhances their marital relationship.

Compared with other countries, the EPDS scores in this study were higher than those of research in Ethiopia (23.7% more than 7 total EPDS scores) and Nigeria (18.6% and 14.6% more than 8 total EPDS scores) [9, 23, 24]. In addition, the EPDS scores of mothers supported by single-VHVs were higher in Thailand (16.8% more than 9 total EPDS scores), Indonesia (22.4% more than 10 total EPDS scores), and India (11.9% more than 12 total EPDS scores) [25–27]. However, the results were lower than those of studies conducted in Pakistan (36% more than 11 total EPDS scores) [28].

This study was implemented in rural areas and used community-based data collection, similar to research in India and Pakistan [27, 28]. However, the studies in Ethiopia, Nigeria, Thailand, and Indonesia involved hospital or clinical based data collection [9, 23–26]. Thus, only mothers who had access to facility-based health services were targeted, which might have influenced their better EPDS scores compared to the participants in this study, especially the mothers supported by single-VHVs. These results may be influenced by the study setting and methodological differences, although the mental health status of participants, especially of mothers supported by single-VHVs, was relatively worse, even among other LMICs. Therefore, expanding the pair-VHV program is very important for ethnic minority women in remote rural areas in Lao PDR.

### Limitations

This study highlights the need for a focus on ethnic minority mothers living in remote and rural villages in Lao PDR. However, this study has certain limitations. First, this was a cross-sectional posttest design study with nonequivalent groups. Absence of data before the introduction of pair-VHVs in the villages is a limitation. The difference between two groups in this study could therefore be attributed to selection differences at baseline. There is therefore the possibility that mothers supported by pair-VHVs possessed better mental health status before the project started.

Second, this study focused on the following factors: (1) biological characteristics, (2) economic status, and (3) social factors. However, other factors such as psychological, obstetric, and pediatric factors are also associated with postnatal depressive symptoms [29]. This information is difficult to collect in this study population. However, further clinical research will likely show improvements in postnatal depressive symptoms.

Finally, this study covers all villages where VHVs work as pairs. Comparative villages were selected where VHVs work alone in the same district. However, the participation rate was lower in villages where VHVs worked as pairs (64.2%) than in villages where they worked alone (91.3%). The number of targets for this analysis was only 34 in villages where VHVs work alone and 86 in villages where they work as pairs (29.6% and 45.3% of the total target population, respectively). This difference may have influenced the results of this study. Examining inter-interviewer differences is also necessary but was not possible in this study due to the limited numbers of participants. Despite these limitations, this study is valuable as it highlights the need for a focus on ethnic minority mothers living in remote and rural villages in Lao PDR where it is difficult to access essential and basic health services.

## Conclusions

Mothers supported by pair-VHVs had a significantly lower EPDS score than those supported by single-VHVs in a rural district of Lao PDR. The findings of this study suggest that the support of male and female health volunteer pairs contributes to improving the mental health status of ethnic minority mothers in remote rural areas in Lao PDR where it is difficult to access essential and basic health services. The expansion of a program to train female VHVs to work with male VHVs is necessary for improving maternal and child health in the area.

## Data Availability

The datasets used and/or analyzed during the current study are available from the corresponding author on reasonable request.

## Abbreviations

ANC: Antenatal care
CHWs: Community health workers
EPDS: Edinburgh Postnatal Depression Scale
HICs: High-income countries
LMICs: Low- and middle-income countries
PDR: People’s Democratic Republic
VHVs: Village health volunteers
WHO: World Health Organization
